# The Sunny Side of Negative Feedback: Negative Feedback Enhances One’s Motivation to Win in Another Activity

**DOI:** 10.3389/fnhum.2021.618895

**Published:** 2021-08-12

**Authors:** Hui Fang, Ximei Li, Haiying Ma, Huijian Fu

**Affiliations:** ^1^School of Business Administration, Guangdong University of Finance, Guangzhou, China; ^2^Laboratory of Neuromanagement and Decision Neuroscience, Guangdong University of Technology, Guangzhou, China; ^3^School of Management, Guangdong University of Technology, Guangzhou, China

**Keywords:** negative feedback, self-determination theory, competence frustration, event-related potentials, reward positivity, restorative process

## Abstract

Negative feedback has been widely reported to be a demotivator that could frustrate the recipient’s need for competence and erode his intrinsic motivation in the same activity. Nevertheless, little attention has been devoted to the intertemporal effect of negative feedback on one’s intrinsic motivation in another activity. To fill this gap, we arranged participants in a game with two sessions and manipulated the content of feedback as a between-subject factor. In session 1, participants had to complete a time estimation task with moderate difficulty, during which half of the participants received normal performance feedback and the other half received negative performance feedback. In session 2, all participants were guided to accomplish a moderately difficult stopwatch task that was competence-supportive. A more pronounced win-loss difference wave of reward positivity (RewP) was detected in the experimental (negative performance feedback) group compared to the control (normal performance feedback) group during session 2. This finding indicates that negative feedback in an activity may have a positive impact on one’s intrinsic motivation in a following competence-supportive activity.

## Introduction

Feedback contains competence-related information regarding one’s performance or ability on a task and can be divided into positive feedback and negative feedback by valence. Receiving feedback about our action, which is common in our daily lives, greatly contributes to our learning, motivation and self-awareness (Narciss, 2004; Wulf et al., 2010). However, individuals are more likely to embrace positive feedback than negative feedback because positive feedback is self-affirming and helps to boost self-efficiency and confidence, whereas negative feedback is self-threatening and may dampen ones’ confidence (Audia and Locke, 2003). It is suggested that negative feedback commonly has a detrimental effect on emotion (Bernichon et al., 2003), job performance (Kluger and DeNisi, 1996), intrinsic motivation (Jussim et al., 1995), and group cooperation (Peterson and Behfar, 2003) in the same activity. Though the negative effect brought by negative feedback in an activity has been extensively studied, the influence of negative feedback on a subsequent activity has yet received little attention. In fact, individuals are engaged in multiple activities in a row in many cases (e.g., in the workplace), rendering the cross-activity effect of negative feedback a prominent issue to be addressed. Therefore, the primary aim of the present study is to investigate the intertemporal effect of negative feedback on the recipient’s intrinsic motivation in a following activity.

According to the self-determination theory (SDT), feeling of competence is a basic psychological need closely associated with personal growth, motivation, internalization and psychological well-being (Deci and Ryan, 2000). Within SDT, the social context such as the provision of negative feedback based on social comparison may thwart an individual’s feeling of competence (Ryan and Deci, 2017). A recent meta-analysis indicates that, compared with positive feedback, negative feedback predicts a higher degree of competence frustration (Fong et al., 2018). Competence frustration refers to the feelings of inadequacy or failure and the doubt of one’s own abilities (Bartholomew et al., 2011). For example, when you repeatedly modify the design scheme and yet it is still disproved by your supervisor, you will have the feeling of failure and begin to doubt whether you are competent for the job. Additionally, a host of studies have demonstrated that need frustration predicts ill-being (Bartholomew et al., 2014), counterproductive work behavior (Van Den Broeck et al., 2014) and disengagement (Jang et al., 2016).

Despite the negative effect caused by the basic psychological need frustration, it is suggested that need frustration might also activate a restorative process (Fiske, 2004; Veltkamp et al., 2009; Radel et al., 2011, 2014). For example, individuals who suffer autonomy frustration in a task would pay more attention to autonomy-related stimuli (Radel et al., 2011) and show increased intrinsic motivation (Radel et al., 2014) in a subsequent task, as the latter task benefits them in restoring frustrated autonomy. Similarly, competence-frustrated individuals are more sensitive to competence-related cues and show a stronger attentional bias to competence-related words in the following dot probe task (Waterschoot et al., 2019). Therefore, there exists a restorative process for competence frustration. In line with these studies, we predict that negative feedback that frustrates one’s competence need in an activity might have a positive impact on intrinsic motivation in a following activity.

To test the hypothesis, we employed a between-subject experimental design in the current study. Event-related potentials (ERPs) method was used to measure intrinsic motivation given that one’s intrinsic motivation is an internal psychological response and its measurement could be easily biased by self-report method. To be specific, reward positivity (RewP), a representative component of ERPs typically detected in the process of feedback and outcome evaluation (Miltner et al., 1997), was used as a candidate neural index for intrinsic motivation (Meng and Ma, 2015; Meng et al., 2016; Fang et al., 2018). RewP is sensitive to the motivational and affective significance of an outcome and the win-loss difference wave of RewP (i.e., the mean amplitude elicited by winning condition minus that by losing condition) reflects a swift subjective evaluation about motivational significance (Gehring and Willoughby, 2002; Yeung et al., 2005). In accordance with existing literature (Ma et al., 2014b; Meng and Ma, 2015; Fang et al., 2018, 2019), we adopted RewP as a neural indicator of intrinsic motivation. We predict that the positive impact of negative feedback on intrinsic motivation in a subsequent activity would be represented by a larger difference wave of RewP in the experimental group relative to the control one.

## Materials and Methods

### Participants

Before we started this experiment, we conducted a power analysis with a medium effect size (*F* = 0.4) and an error probability (α) of 0.05 to figure out the proper sample size. The suggested sample size is 44. Accordingly, we recruited 46 healthy right-handed participants from a university in southern China in the present study. Participants were randomly arranged in the control group (*N* = 23, 10 females) or the experimental group (*N* = 23, 10 females). All of them had either normal or corrected-to-normal vision, and reported to be free of any history of neurological disorders or mental diseases. Before the formal experiment, we obtained written informed consent from each of the participants. The Internal Review Board of the Laboratory of Neuromanagement and Decision Neuroscience from Guangdong University examined and approved the experimental protocol. Participants were not informed of the true purpose of the study during the experiment, but they were debriefed about the purpose after the experiment.

### Experimental Paradigms

**FIGURE 1:**
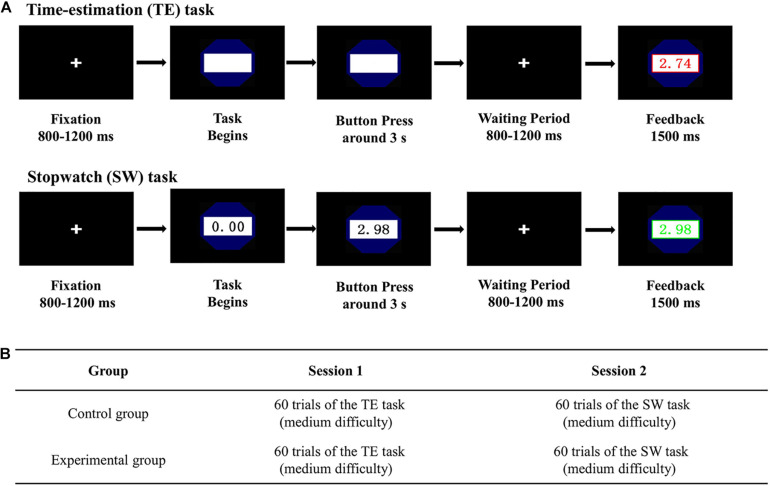
Demonstration of the experimental paradigm. **(A)** TE and SW tasks. **(B)** The experimental procedure.

Participants were comfortably seated in a dimly lit, sound-attenuated and electrically shielded apartment. We presented all experimental stimuli (6.2° × 5.4° of visual angle) at the center of a computer screen 100 cm away from the participants. Participants were told to complete two different tasks in sequence and instructed to use a keypad to make responses prior to the experiment. As depicted in Figure 1B, the experiment consisted of 2 sessions, each of which contained 60 trials. All participants had to complete a TE task of moderate difficulty [which had a success interval of (2.75 s, 3.25 s)] in session 1. Following that, they were required to complete a SW task of moderate difficulty [which had a success interval of (2.93 s, 3.07 s)] in session 2. The pre-defined success time intervals for the two tasks were identical with one of our recent studies (Fang et al., 2018), in which a pilot study was performed to determine the appropriate time windows. Participants were told that the whole experiment included 2 sessions at the beginning. Before each session began, we guided the participants to read the corresponding instruction and asked them to complete several practice trials to get familiar with the task. E-Prime 2.0 software (Psychology Software Tools, Pittsburgh, PA, United States) was used to display the stimuli, and record triggers and behavioral responses.

By the end of session 2, participants were required to assess their perceived competence frustration in session 1 on 7-point scales, with 1 indicating “do not fully agree” and 7 indicating “totally agree”. The scale was developed from extant literature (Chen et al., 2015; Schultz et al., 2015). Sample items are “I have serious doubts about whether I can play the time-estimation game well” and “I feel disappointed with my performance in the time-estimation game” (α = 0.763). The participants were debriefed and paid in the end.

#### The Time-Estimation (TE) Task

For the TE task, the goal for all participants was to estimate time durations of 3 s, and the closer of the estimate to the target, the better (Meng and Ma, 2015). As illustrated in Figure 1A, for each trial, a cross icon was firstly displayed for 800–1,200 ms. Then a still stopwatch pattern appeared, suggesting the starting point of time estimation. If participants thought that 3 s had passed away, they needed to immediately press any single button on the keypad. Following the reaction, a cross icon was shown again for 800–1,200 ms. After that, the specific stop time (feedback information) would be presented for 1,500 ms. If the reaction dropped in the appointed success time range, the stop time would be showed in green. Otherwise, it would be shown in red. Finally, there was a randomized blank interval which lasted for 600–1,000 ms before the next trial started.

#### The Stop-Watch (SW) Task

For the SW game, the goal for all participants was to cease a going watch at around 3 s. Identically, the closer of the stop time to the target, the better (Murayama et al., 2010; Albrecht et al., 2014; Ma et al., 2014a). The procedure of the SW task was similar to that of the TE task except that there was a going stopwatch. Specially, as shown in Figure 1A, for each trial, a cross icon was firstly displayed for 800–1,200 ms. Afterward, a stopwatch icon would appear and start running automatically. Participants were asked to cease it at as close to 3 s as possible. The feedback paradigm was identical with the TE task in session 1.

#### Experimental Manipulation

Prior research has suggested that negative feedback that focuses on social comparison or normative standards usually lead to negative outcomes (Bernichon et al., 2003; Peterson and Behfar, 2003; Van Dijk and Kluger, 2011) and may bring competence frustration for one that receives the negative feedback (Ryan and Deci, 2017). Consequently, in the present study, negative feedback based on social comparison was provided for participants in the experimental group during session 1 in order to successfully induce competence frustration among them. Specifically, participants in the control group received normal performance feedback while those in the experimental group obtained normal performance feedback and an additional negative feedback. Each time after completing 10 trials, participants in the experimental group would receive negative feedback concerning the past 10 trials, which incorporated a planned short text and a fictitious ranking evaluation. To be specific, the planned short text was “Your performance was below average,” and the fictitious ranking evaluation was presented as “You performed worse than x% of the participants in the latest 10 rounds.” The value of x fluctuated between 50 and 100 depending on participants’ actual performance for the sake of increasing the plausibility of the feedback. Unknown to those participants, their rankings were systematically underestimated in the experiment. Participants in the control group only received normal performance feedback after each trial, i.e., “If the reaction dropped in the appointed interval, the stop time will be presented in green. If not, in red.”

### Electroencephalogram (EEG) Data Recordings

Behavioral data were recorded by E-Prime 2.0 software (Psychology Software Tools, Pittsburgh, PA, United States). EEG data were recorded by ego amplifier together with a Waveguard EEG Cap mounted with 64 Ag/AgCl electrodes (produced by ANT Neuro, Enschede, Netherlands). The channel data went through online band-pass-filter from 0.1 to 100 Hz and were recorded at a sampling rate of 500 Hz. The EEG experiment would start when all electrode impedances were decreased to below 10 kΩ and remained stable. We employed the left mastoid as online reference electrode during the experiment, and computed the average value of the left and right mastoids for off-line re-referencing.

### Data Reduction

ASALab 4.10.1 software package (ANT Neuro, Enschede, Netherlands) was applied for off-line EEG data analyses. The procedures were as follows: (a) a digital low-pass filter at 30 Hz (24 dB/octave); (b) identification and correction of ocular artifacts using a principle component analysis-based eye movement correction algorithm embedded in the ASALab program; (c) segmentation of −200/+800 ms upon feedback stimuli onset; (d) baseline correction with the 200 ms pre-stimuli interval serving as baseline; (e) artifact detection by which trials involving amplifier clippings, bursts of electromyography activity, or peak-to-peak deflections that exceeded ± 100 μV were removed from averaging; and (f) averaging across all trials within each condition. Specially, for each participant, the recorded EEGs over each recording site were averaged for each feedback type (including the winning condition and losing condition). Moreover, computing a difference waveform is recommended for examining the FRN/RewP (Meng and Ma, 2015; Fang et al., 2019), since there is nothing special about the local maxima and minima on the conditional waveforms (Luck, 2005). Consequently, the win-lose difference wave was also computed in the present study.

### Data Analysis

For the behavioral data, an independent sample *t*-test was used to compare the perceived competence frustration in session 1 between the experimental and control groups. Similarly, the success rates and mean errors for session 1 and session 2 were analyzed separately by independent sample *t*-tests. It has to be noted that success rate is defined as the percentage of responses that fall within the success time interval, and mean error is defined as the absolute discrepancy value between the reaction time and the target time (i.e., 3 s).

As to the ERP data, we mainly focused on RewP and P300, which were demonstrated to be closely related with feedback processing and outcome evaluation (Proudfit, 2015; Muhlberger et al., 2017). RewP is indicative of the motivational and/or affective significance of the outcome, and the win-loss difference wave of RewP reflects a swift subjective evaluation about motivational significance (Masaki et al., 2006; Meng and Ma, 2015; Meng et al., 2016; Fang et al., 2019). The amplitude of RewP generally reaches its maximum during 250–300 ms after feedback onset. Based on the visual inspection of the grand averaged waveforms as well as prior research, the mean voltage within 220–290 ms was calculated for RewP analysis in this study. Extant literature suggests that RewP is most pronounced at fronto-central electrodes, such as FZ, FCZ, and CZ (Ullsperger et al., 2014; Oemisch et al., 2017; Fernandes et al., 2018; San Martin, 2012), which is also true in the current study. Meanwhile, in line with existing literature (Leng and Zhou, 2009; Wu and Zhou, 2009; Jones et al., 2012), the mean voltage within 290–400 ms over the central-parietal and parietal electrodes (including CP1, CPZ, CP2, P1, PZ, P2) was computed for P300 analysis. As electrode is not suggested to be included as an additional factor during statistical analyses (Luck and Gaspelin, 2017), the mean voltages were averaged across the electrode cluster for RewP (F1, FZ, F2, FC1, FCZ, FC2, C1,CZ, C2) before being submitted to a repeated measure ANOVA. Similarly, the mean voltages were averaged across the electrode cluster for P300 (CP1, CPZ, CP2, P1, PZ, P2) before further analysis.

## Results

Participants of the experiment ranged in age between 19 and 23 years (*M* = 19.70, *SD* = 0.87). Two participants with excessive artifacts were excluded; thus, data from 22 valid participants in the control group (10 females) and 22 valid participants (10 females) in the experimental group were used for the final analysis.

### Behavioral Results

The independent sample *t*-test showed that participants in the experimental group (*M* = 4.511, *SD* = 1.081) experienced a higher level of competence frustration compared with those in the control group [*M* = 3.057, *SD* = 1.270, *t*(42) = −4.090, *p* < 0.001, Cohen’ s *d* = 1.233] when working on the TE task during session 1.

However, there were no significant between-group differences in success rates [*M*_control_ = 0.610, *SD* = 0.149; *M*_experimental_ = 0.546, *SD* = 0.106; *t*(42) = −1.670, *p* = 0.102, Cohen’ s *d* = 0.495] and mean errors [*M*_control_ = 0.255, *SD* = 0.078; *M*_experimental_ = 0.291, *SD* = 0.061; *t*(42) = 1.651, *p* = 0.106, Cohen’ s *d* = −0.514] of the TE task during session 1. Likewise, the success rates [*M*_control_ = 0.504, *SD* = 0.131; *M*_experimental_ = 0.478, *SD* = 0.102; *t*(42) = 0.748, *p* = 0.459, Cohen’ s *d* = 0.221] and mean errors [*M*_control_ = 0.097, *SD* = 0.037; *M*_experimental_ = 0.093, *SD* = 0.021; *t*(42) = 0.427, *p* = 0.672, Cohen’ s *d* = 0.133] of the SW task in session 2 were not significantly different between the two groups.

### ERP Results

#### RewP

**FIGURE 2:**
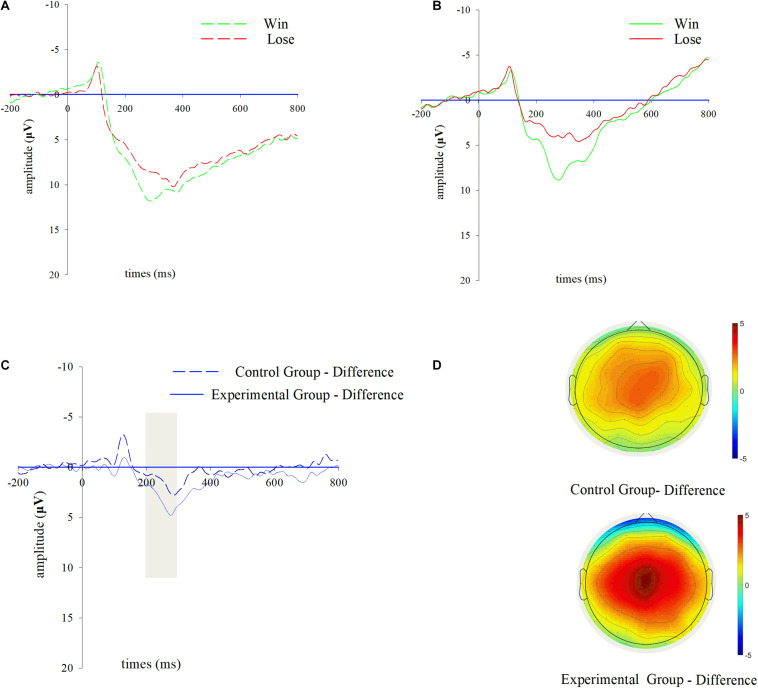
RewP results during outcome evaluation. Grand-averaged ERP waveforms of RewP for wins and losses from the chosen electrode cluster (F1, FZ, F2, FC1, FCZ, FC2, C1, CZ, C2) for **(A)** the control group, and **(B)** the experimental group. **(C)** Difference waves of RewP (win-lose) from the chosen electrode cluster for two groups. **(D)** The scalp topographies of difference waves of RewP for two groups, and the bar for the topographic map ranges from –5 to 5 μV.

The grand averaged waveforms for RewP in the electrode cluster (F1, FZ, F2, FC1, FCZ, FC2, C1, CZ, C2) are displayed in Figure 2. The mean RewP amplitudes in the given time window were 7.405 μV (experimental group-win), 3.734 μV (experimental group-lose), 10.041 μV (control group-win) and 8.250 μV (control group-lose) in respective conditions. A 2 (group: control, experimental) × 2 (outcome: win, lose) mixed model repeated measure ANOVA was employed on RewP amplitudes. The results indicated a significant main effect of outcome [*F*_(1,42)_ = 45.597; *p* < 0.001; η^2^ = 0.521] and group [*F*_(1,42)_ = 7.263; *p* = 0.01; η^2^ = 0.147]. The main effect of outcome revealed that the RewP was more positive in the winning condition than in the losing one. Moreover, the interaction effect between group and outcome was significant [*F*_(1,42)_ = 5.406; *p* = 0.025; η^2^ = 0.114], suggesting that the difference wave of RewP was markedly larger in the experimental group (*M* = 3.671, *SD* = 0.56) than in the control group (*M* = 1.791, *SD* = 0.58). In addition, simple effect analyses revealed more pronounced RewP amplitude in the winning condition than in the losing one in both the experimental group [*F*_(1,21)_ = 30.847; *p* < 0.001; η^2^ = 0.595] and the control group [*F*_(1,21)_ = 14.753; *p* < 0.001; η^2^ = 0.413].

#### P300

**FIGURE 3:**
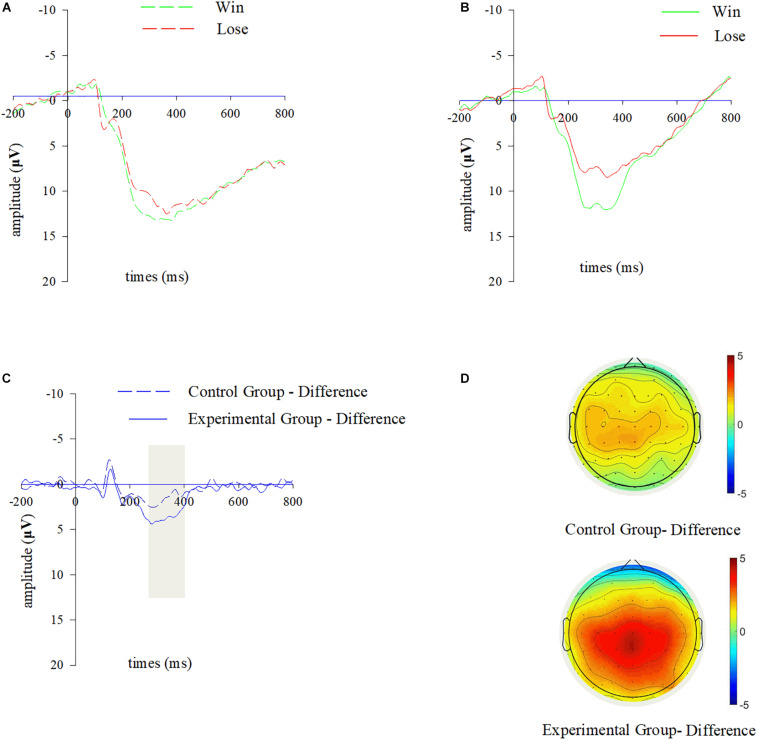
P300 results during outcome evaluation. Grand-averaged ERP waveforms of P300 for wins and losses from the chosen electrode cluster (CP1, CPZ, CP2, P1, PZ, P2) for **(A)** the control group, and **(B)** the experimental group. **(C)** Difference waves of P300 (win-lose) from the chosen electrode cluster for two groups. **(D)** The scalp topographies of difference waves of P300 for two groups, and the bar for the topographic map ranges from –5 to 5 μV.

A 2 (group: control, experimental) × 2 (outcome: win, lose) mixed model repeated measure ANOVA for P300 amplitudes indicated a significant main effect of outcome [*F*_(1,42)_ = 19.447; *p* < 0.001; η^2^ = 0.316]. P300 was more positive in the winning condition (*M* = 11.711 μV, *SD* = 0.767) than in the losing one (*M* = 9.534 μV, *SD* = 066). However, the main effect of group did not reach statistical significance [*F*_(1,42)_ = 3.153; *p* = 0.083; η^2^ = 0.07], indicating no between-group difference on P300 amplitudes. The interaction effect between group and outcome was not significant [*F*_(1,42)_ = 2.807; *p* = 0.101; η^2^ = 0.063], demonstrating that the difference wave of P300 did not differ between the experimental and control groups (see Figure 3).

## Discussion

Negative feedback is prevalent in our daily lives, as we might receive negative feedback about our performance or ability from those around us, such as our friends, teachers, superiors, and colleagues. Hence considerable attention has been paid to the consequences caused by negative feedback. It has been demonstrated that negative feedback has a negative effect on intrinsic motivation, and this effect is modulated by the characteristics and context of feedback (Fong et al., 2018). However, uncertainty remains as to whether the prior negative feedback has a spillover effect on intrinsic motivation in a following activity. Thus, the present study attempts to gain more insight into this issue by engaging participants in a two-session game with their scalp EEG recorded simultaneously.

### Research Findings

Participants were instructed to complete a TE task in session 1 and a SW task in session 2, both of which were moderately difficult and competence-supportive. A between-subject design was adopted such that participants in the experimental group received negative feedback about their performances while those in the control group only received normal performance feedback. As expected, we observed a more pronounced difference wave of RewP in the experimental group than in the control group in session 2. Based on the motivational significance theory, the difference wave of RewP reflects a swift subjective evaluation of the motivational significance (Gehring and Willoughby, 2002; Yeung et al., 2005). When an outcome feedback in a specified condition conveys a higher level of motivational significance, the difference wave of RewP will be augmented (Masaki et al., 2006; Ma et al., 2014b; Meng and Ma, 2015; Meng et al., 2016). Take Ma et al. (2014b)’s study as an example, the authors engaged participants in tasks with different levels of difficulty (multiplications vs. additions) and noted that the more effort-requiring task (multiplications) increased the motivational significance of receiving the performance feedback, which was reflected by a larger difference wave of RewP. Moreover, some recent electrophysiological studies have proposed the amplitude of RewP upon feedback to represent one’s intrinsic motivation either when external bonus is not paid or when show-up fee is uncorrelated with task performance (Ma et al., 2014b; Meng and Ma, 2015). Accordingly, our finding might suggest that the task performances bore more motivational significance to participants who received negative feedback in a prior task. Participants were more motivated to achieve good performance in session 2 after receiving negative feedback in session 1. Therefore, this finding implies that prior negative feedback has a potentially positive influence on one’s intrinsic motivation to win in a following activity. It’s also noteworthy that unlike RewP, the difference waves of P300 did not show any difference between the experimental group and the control group, suggesting that RewP and P300 might embody different cognitive processes and RewP was more sensitive to the motivational significance of task performance after participants received negative performance feedback in a prior task.

Though negative feedback has been found to have a detrimental influence on intrinsic motivation in the same activity, this work indicates a reversed relationship between prior negative feedback and intrinsic motivation in a following activity. The finding of this study seems counter-intuitive, but the restoration process evoked by competence frustration might account for this effect. Results obtained from subjective ratings showed that, compared to participants in the control group, those in the experimental group suffered a higher level of competence frustration. A recent study suggests that there exists a competence restoration process when need for competence is frustrated. Specially, individuals who suffer competence frustration allocate more attention to competence-related stimuli in the following activity, because these stimuli help them restore frustrated competence (Waterschoot et al., 2019). In accordance with extant literature, we speculate that the positive impact of negative feedback on intrinsic motivation in the subsequent activity might be due to that the subsequent new activity provides a chance for the individuals to restore the feeling of competence that is frustrated in the former task. Thus, our findings provides support for the previous literature with electrophysiological evidences (Fong et al., 2018).

### Theoretical and Practical Significance

Theoretically, this study extends existing literature on intrinsic motivation. Previous studies on intrinsic motivation have been mainly focused on the effects of material motivation (Eckel, 1999; Vansteenkiste and Deci, 2003), spirit motivation (Deci et al., 1999; Avila et al., 2012; Albrecht et al., 2014) and job characteristics (Gagné, 2014; Meng and Ma, 2015) on intrinsic motivation. As far as we know, this is the initial study that delves into the intertemporal impact of negative feedback on one’s intrinsic motivation in a following competence-supportive task. Our results suggest that prior negative feedback may increase one’s intrinsic motivation (reflected by RewP) in a subsequent task that brings a sense of competence.

Based on the above finding, this study also has implications for managerial practice. In many cases, negative feedback is unavoidable in the workplace and is highly likely to cause the employees’ need for competence to be frustrated. However, there is a tendency for them to take actions to regain competence. As a result, managers should guarantee that the following work is competence-supportive. Such work arrangement provides the employees a chance to enhance their intrinsic motivation and restore competence. Importantly, though negative feedback might activate a competence-restorative process in a subsequent activity, we do not encourage the managers to arbitrarily provide negative feedback to employees. Feeling of competence is critical for one’s subjective well-being. In order to satisfy the employees’ need for competence, it is of great importance for managers to reduce the use of negative feedback based on social comparison and to provide more positive feedback to them.

### Limitations and Future Research

Although the present research provides initial neural evidence for the potential positive intertemporal effect of negative feedback on one’s intrinsic motivation, it also has some limitations that are worthy of attention. First, we resorted to RewP as a neural indicator of intrinsic motivation, but we did not measure intrinsic motivation by self-report. It is highly recommended that future research should use multiple methods to measure intrinsic motivation to provide further support for the current study. Second, the direction of feedback flow might influence the consequence of negative feedback (Kim and Kim, 2019). It is an interesting issue for future study to explore if prior negative feedbacks received from different agents (e.g., superior, subordinate and colleague) have different effects on intrinsic motivation. Third, this study employed two highly related tasks (time estimation task and stopwatch task) in sequential sessions, which limited the generalizability of the research findings to contexts with less related tasks. Future research is warranted to examine if the findings of the present study hold when less related tasks are used. Fourth, recent research suggests that there may be individual difference in handling negative feedback and one’s feedback control system would impact the coping style following negative feedback (Franklin and Frank, 2015). It is a promising issue to explore the individual factors that may modulate the intertemporal effect of negative feedback on one’s intrinsic motivation.

## Conclusion

The current study aims to uncover the effect of negative feedback on intrinsic motivation in the subsequent activity. Electrophysiological data suggests that participants who received negative feedback (vs. those who did not) suffered a greater level of competence frustration in the former task and showed heightened intrinsic motivation in the subsequent competence-supportive activity (as reflected by an augmented difference wave of RewP). Our findings supplement existing literature on the feedback-motivation relationship and provide important practical implications for managers.

## Data Availability Statement

The raw data supporting the conclusions of this article will be made available by the authors, without undue reservation.

## Ethics Statement

The studies involving human participants were reviewed and approved by Internal Review Board of the Laboratory of Neuromanagement and Decision Neuroscience in Guangdong University of Technology. The patients/participants provided their written informed consent to participate in this study.

## Author Contributions

HFu, HF, and XL conceived, designed the study, interpreted the data, and drafted the manuscript. HF collected and analyzed the data. HFu, HF, XL, and HM reviewed and edited the manuscript. HFu administered the project. All authors contributed to the article and approved the submitted version.

## Conflict of Interest

The authors declare that the research was conducted in the absence of any commercial or financial relationships that could be construed as a potential conflict of interest.

## Publisher’s Note

All claims expressed in this article are solely those of the authors and do not necessarily represent those of their affiliated organizations, or those of the publisher, the editors and the reviewers. Any product that may be evaluated in this article, or claim that may be made by its manufacturer, is not guaranteed or endorsed by the publisher.
